# An accessory wall teichoic acid glycosyltransferase protects *Staphylococcus aureus* from the lytic activity of *Podoviridae*

**DOI:** 10.1038/srep17219

**Published:** 2015-11-24

**Authors:** Xuehua Li, David Gerlach, Xin Du, Jesper Larsen, Marc Stegger, Petra Kühner, Andreas Peschel, Guoqing Xia, Volker Winstel

**Affiliations:** 1 Infection Biology, Interfaculty Institute of Microbiology and Infection Medicine, University of Tübingen, Auf der Morgenstelle 28, 72076 Tübingen, Germany; 2 German Center for Infection Research (DZIF), partner site Tübingen, 72076 Tübingen, Germany; 3 Microbiology and Infection Control, Statens Serum Institut, Artillerivej 5, 2300 Copenhagen, Denmark; 4 Pathogen Genomics Division, Translational Genomics Research Institute, 3051 W Shamrell Blvd, Flagstaff, 86001 Arizona, USA; 5 Institute of Inflammation & Repair, The University of Manchester, Manchester, United Kingdom

## Abstract

Many *Staphylococcus aureus* have lost a major genetic barrier against phage infection, termed clustered regularly interspaced palindromic repeats (CRISPR/cas). Hence, *S. aureus* strains frequently exchange genetic material via phage-mediated horizontal gene transfer events, but, in turn, are vulnerable in particular to lytic phages. Here, a novel strategy of *S. aureus* is described, which protects *S. aureus* against the lytic activity of *Podoviridae*, a unique family of staphylococcal lytic phages with short, non-contractile tails. Unlike most staphylococcal phages, *Podoviridae* require a precise wall teichoic acid (WTA) glycosylation pattern for infection. Notably, TarM-mediated WTA α-O-GlcNAcylation prevents infection of *Podoviridae* while TarS-mediated WTA β-O-GlcNAcylation is required for *S. aureus* susceptibility to podoviruses. Tracking the evolution of TarM revealed an ancient origin in other staphylococci and vertical inheritance during *S. aureus* evolution. However, certain phylogenetic branches have lost *tarM* during evolution, which rendered them podovirus-susceptible. Accordingly, lack of *tarM* correlates with podovirus susceptibility and can be converted into a podovirus-resistant phenotype upon ectopic expression of *tarM* indicating that a “glyco-switch” of WTA O-GlcNAcylation can prevent the infection by certain staphylococcal phages. Since lytic staphylococcal phages are considered as anti-*S. aureus* agents, these data may help to establish valuable strategies for treatment of infections.

Horizontal gene transfer (HGT) events are prerequisites for bacterial evolution. Bacteria, including many Gram-positive pathogens, employ different mechanisms for the exchange of genetic information. Major mechanisms include bacteriophage- (phage) mediated transduction, conjugation, and transformation^1^,^2^. These factors substantially contribute to bacterial evolution but vary in their impact depending on the bacterial species.

During evolution, many bacteria evolved various protective mechanisms that interfere with or impede HGT events. “Clustered regularly interspaced palindromic repeats” (CRISPR/cas) loci, for example, recognize invading DNA and confer bacterial adaptive immunity to phage infection^3^. Other strategies to avoid HGT include restriction modification (R-M) systems, which most likely evolved in order to avoid uptake of foreign DNA from sources other than the same or related bacterial species^1^,^4^,^5^,^6^. However, in many pathogenic bacteria including the major human pathogen *Staphylococcus aureus*, particular phage-mediated transduction is probably the most efficient and important mechanism to exchange genetic information^7^,^8^. Typically, *S. aureus* benefits from phage-mediated HGT events, since many staphylococcal phages mobilize resistance plasmids, genomic islands or other genomic loci with determinants of bacterial virulence^9^,^10^, thus substantially contributing to the evolution, pathogenicity, and global spread of this pathogen. Hence, protective mechanisms, which interfere with or even completely prevent phage infection and phage-mediated HGT events, can appear disadvantageous and maintain pathogens such as *S. aureus* in an evolutionary “dead-end”. Such a scenario is probably a reason for the emergence of phylogenetically isolated branches, as reported recently for the unique *S. aureus* lineage sequence type (ST) 395, which completely changed the phage adsorption receptor properties rendering it resistant from HGT with other *S. aureus* lineages^11^,^12^. However, such dramatic changes in the phage receptor properties are probably very rare among *S. aureus* clones and do not represent a frequent strategy to prevent phage adsorption or other phage-mediated HGT events.

Apart from ST395 isolates, which synthesize a unique glycerol-phosphate (GroP) WTA substituted with D-alanine and α-O-N-Acetylgalactosamine (GalNAc)^11^,^12^, most *S. aureus* clones synthesize a ribitol-phosphate (RboP) WTA repeating unit substituted with three tailoring modifications, D-alanine, α-O-N-acetylglucosamine (GlcNAc), and β-O-GlcNAc^13^,^14^. The GlcNAc moieties are attached to RboP by two independent enzymes, the α-O-GlcNAc WTA glycosyltransferase TarM^15^, and the β-O-GlcNAc WTA transferase TarS^16^. Most *S. aureus* phages and phage-related *S. aureus* pathogenicity island (SaPI) particles target these WTA O-GlcNAc moieties for adsorption and subsequent infection^11^,^15^,^16^,^17^. Apparently, the stereochemical linkage of WTA glycosylation is dispensable for the phage infection process since strains lacking one of the two WTA glycosyltransferases are still phage or SaPI-particle susceptible^11^,^16^. In contrast, staphylococcal *Myoviridae* simply require WTA polymers, regardless of the polyol type or WTA O-GlcNAcylation^11^,^12^,^17^. Nevertheless, since WTA polymers have many other crucial functions in *S. aureus* pathogenesis and resistance^13^,^14^, most staphylococcal phages seem to be well-adapted to a rather conserved and important cell surface molecule, which *S. aureus* presumably does not mutate frequently. Accordingly, phage infection-preventing mutations in WTA biosynthesis genes have not been described so far. Thus, phage-mediated HGT events among *S. aureus* clones frequently occur and are rather beneficial for *S. aureus* evolution and adaptation to changing selection pressures, which is, conversely, also supported by the notion that many *S. aureus* clones if not all (as suggested by a recent *in silico* study^18^) have lost CRISPR/cas loci, which otherwise disable or even completely block HGT. Accordingly, staphylococcal phage protection mechanisms most likely evolved to prevent phage lysis, caused by lytic but not by transducing or beneficial phages.

Here, a novel strategy of *S. aureus* is described to prevent adsorption and infection of *Podoviridae*, a specific class of staphylococcal lytic phages with very short, non-contractile tails. This strain-specific barrier, which was lost by various *S. aureus* lineages during evolution, can completely block the *Podoviridae* infection process thereby providing new insights into bacterial strategies to counteract phage infections.

## Results

### Infection of *S. aureus* by *Podoviridae* is strain-dependent

Lytic *S. aureus* phages, for example staphylococcal *Myoviridae*, usually have a broad host-range and can even infect other staphylococcal species^11^,^19^. Accordingly, the broad host-range phages ΦK and Φ812 (*Myoviridae*) infected and lysed nearly all *S. aureus* test strains including strains of dominant MRSA linages, albeit with different potencies (Table 1). However, a collection of another family of lytic staphylococcal phages (*Podoviridae*; here phages Φ44AHJD, Φ66 and ΦP68) failed to infect certain myovirus-susceptible strains, for instance the two American pandemic CA-MRSA clones USA300 (NRS384) and USA400 (MW2), and the HA-MRSA isolate 605, a member of the predominant Asian ST239 lineage (Table 1). Even though some test strains were susceptible to *Podoviridae*, these phages seem to have a narrower host-range than other lytic staphylococcal phages.

Podovirus-susceptible *S. aureus* strains were found among several clonal lineages suggesting that *Podoviridae* probably do not require an ST-specific receptor for adsorption and infection, as reported recently for the *S. aureus* ST395-specific phage Φ187^11^,^12^ (Table 1). In line with this notion, the strains PS44A, PS66, and P68 recommended for propagation of different podoviruses^20^ were found to belong to different, unrelated STs, when they were multi locus sequence-typed (MLST) (Table 1).

Thus, staphylococcal *Podoviridae* have a specific host-range different from that of other lytic staphylococcal phages such as *Myoviridae*.

### Peptidoglycan-anchored surface proteins are dispensable for host specificity of *Podoviridae*

**Figure 1:**
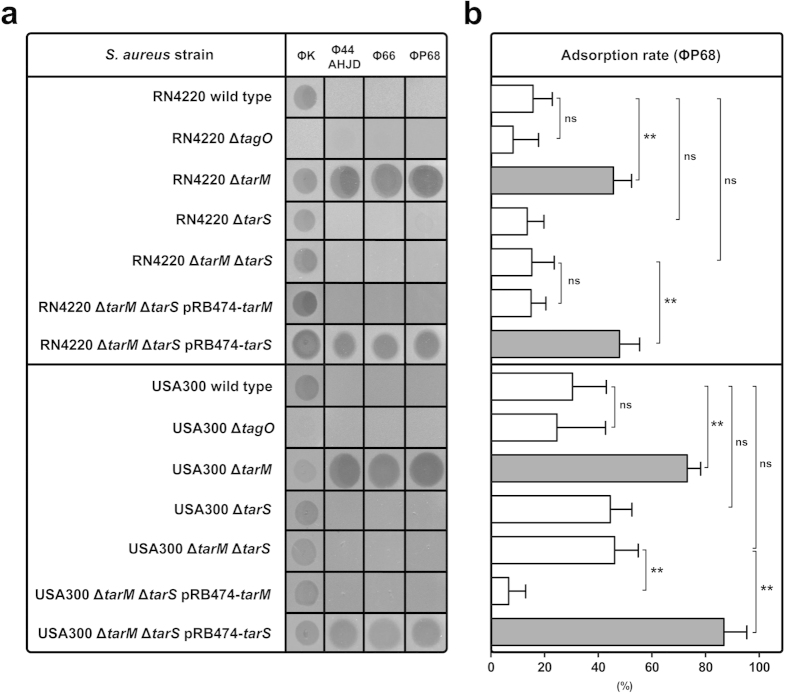
The α-O-GlcNAc WTA glycosyltransferase TarM protects *S. aureus* from the lytic activity of *Podoviridae*. **(a)** *S. aureus* RN4220 and USA300 susceptibility to the broad-host-range lytic phage ΦK (*Myoviridae*), and to the lytic phages Φ44AHJD, Φ66 and ΦP68 (*Podoviridae*) was analyzed using a soft-agar overlay approach. A representative experiment is shown. **(b)** Podovirus ΦP68 adsorption rates (%) to *S. aureus* RN4220 and USA300 variants. *S. aureus* wild type and strains lacking WTA (Δ*tagO*), WTA α-O-GlcNAcylation (Δ*tarM*), WTA β-O-GlcNAcylation (Δ*tarS*), WTA glycosylation (Δ*tarM* Δ*tarS*), and the complemented mutants (Δ*tarM* Δ*tarS* pRB474-*tarM*, Δ*tarM* Δ*tarS* pRB474-*tarS*) are indicated. Values are given as means and standard deviations (SD, n = 3). Statistical significant differences calculated by one-way ANOVA with Bonferroni’s multiple comparison test are indicated: not significant (ns), P > 0.05; *P < 0.05, **P < 0.01.

The specific host-range of *Podoviridae* suggests that these phages might fail to infect and lyse certain *S. aureus* strains due to unique barriers preventing adsorption, infection, or reproduction. Since the commonly used laboratory and podovirus-resistant *S. aureus* strain RN4220 (see Fig. 1 and Supplementary Fig. S1) lacks R-M systems, prophages, and CRISPR/cas loci previously shown to impede HGT, an intracellular barrier facilitating resistance to *Podoviridae* seems implausible. More likely, alterations in peptidoglycan modifications, for example specific cell-surface exposed molecules such as peptidoglycan-anchored ‘microbial surface components recognizing adhesive matrix molecules’ (MSCRAMMs), might block adsorption and infection in certain *S. aureus*. However, *S. aureus* RN4220 mutants and mutants derived from the clinical CA-MRSA isolate USA300 lacking functional surface proteins (Δ*srtA*) were resistant to *Podoviridae* indicating that factors other than MSCRAMMs interfere with the podovirus infection process (Supplementary Fig. S1).

Thus, *S. aureus* peptidoglycan-anchored surface proteins do not influence the unusual host-range of staphylococcal *Podoviridae*.

### The *S. aureus* α-O-GlcNAc WTA glycosyltransferase TarM prevents the lytic activity of *Podoviridae*

Because all studied staphylococcal phages require WTA polymers or O-GlcNAcylated WTA polymers for adsorption and infection^17^, adsorption of *Podoviridae* to their designated cell surface receptors may also be influenced by WTA polymers. Of note, all podovirus-susceptible strains were simultaneously susceptible to the WTA-dependent phages ΦK and Φ812, which excludes that podovirus-susceptible strains fail to produce WTA polymers (Table 1). In line with this assumption, *Podoviridae* still failed to adsorb to and infect *S. aureus* RN4220 or USA300 mutants lacking either WTA (Δ*tagO*) or WTA glycosylation (Δ*tarM* Δ*tarS*) (Fig. 1a,b).

While well-studied WTA-GlcNAc dependent *S. aureus* phages such as phage Φ11 do not seem to require a specific stereochemistry of WTA O-GlcNAc for infection^16^ the tested podoviruses exhibited an unexpected preference for TarS-glycosylated but not TarM-glycosylated WTA. Strikingly, lack of WTA α-O-GlcNAcylation (Δ*tarM*) resulted in dramatically increased binding capacities of phage ΦP68 and rendered strain RN4220 Δ*tarM* highly susceptible to podovirus infection (Fig 1a,b). In contrast, lack of *tarS* did not lead to phage susceptibility of RN4220 (Fig. 1a). Complementation of the WTA-glycosylation deficient Δ*tarM* Δ*tarS* mutant with one of the two *S. aureus* WTA glycosyltransferases TarM or TarS demonstrated that, (i) *Podoviridae* require TarS-mediated WTA β-O-GlcNAcylation, but (ii) are inhibited by TarM-mediated WTA β-O-GlcNAcylation (Fig 1a,b). Similar results were obtained for *S. aureus* USA300 strongly suggesting that TarM diminishes the adsorption and infection of *Podoviridae* to *S. aureus* (Fig. 1a,b). Because TarM is an intracellular protein it appears highly unlikely that it interferes with podovirus binding directly but impedes podovirus binding by α-O-GlcNAcylated WTA.

Thus, the α-O-GlcNAc WTA glycosyltransferase TarM prevents the adsorption and infection by staphylococcal *Podoviridae*.

### Lack of *tarM* correlates with susceptibility to *Podoviridae*

**Figure 2:**
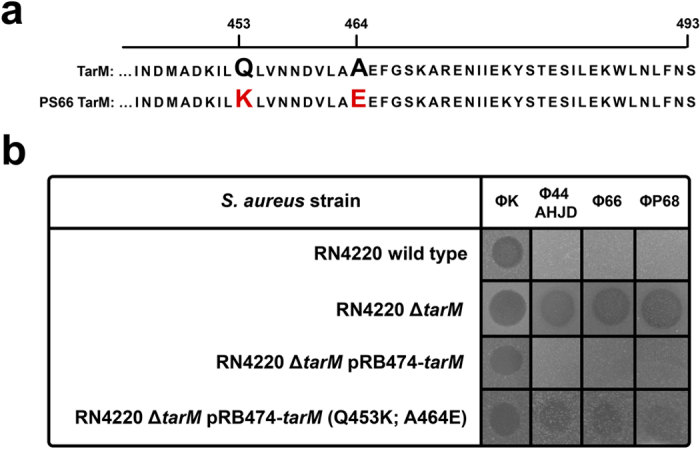
Point mutations in TarM render Φ66 propagation strain PS66 susceptible to *Podoviridae*. **(a)** A sequence alignment of wild-type TarM and PS66 TarM is shown. Position of mutations (Gln-453 with Lys; Ala-464 with Glu) and the end of the open reading frame (493) are indicated. **(b)** *S. aureus* RN4220 susceptibility to the broad host-range lytic phage ΦK (*Myoviridae*), and to the lytic phages Φ44AHJD, Φ66, and ΦP68 (*Podoviridae*) was analyzed using a soft-agar overlay approach. *S. aureus* RN4220 wild type and strains lacking WTA α-O-GlcNAcylation (Δ*tarM*), and the complemented mutants (Δ*tarM* pRB474-*tarM*, Δ*tarM* pRB474-*tarM* (Q453K; A464E) are indicated. A representative experiment is shown.

In order to confirm the inhibitory effect of TarM on podovirus susceptibility, genomes of *S. aureus* test strains were screened for the presence or absence of the genes encoding WTA glycosyltransferases TarM and TarS via PCR or BLASTN of available genomes^21^. Most strains contained *tarS* except for strains PS187, which produce an entirely different type of WTA^11^,^12^, and ED133, which does not encode any of the so far described WTA glycosyltransferases (Table 1). In contrast, several strains lacked *tarM*. As proposed, most *tarM*- plus *tarS*-encoding *S. aureus* strains were podovirus-resistant (Table 1). Conversely, *S. aureus* strains exclusively encoding *tarS* and even other staphylococcal species such as *Staphylococcus xylosus* or *Staphylococcus equorum*, which encode *tarS* homologues with high similarity, but lack *tarM*, were susceptible indicating that *Podoviridae* specifically sense β-O-GlcNAcylated WTA (Table 1 and Supplementary Fig. S2). In line with this, the designated podovirus propagation strains PS44A (Φ44AHJD) and P68 (ΦP68) exclusively encoded *tarS* (Table 1). However, strain PS66 (Φ66) encoded both WTA glycosyltranserases, TarM and TarS, which did not align with the assumption that *tarM* interferes with podovirus susceptibility. Nevertheless, even though *tarM* was expressed at good levels during logarithmic growth phase, *tarS* was significantly higher expressed than *tarM* during early growth stages, which probably promotes the infection by *Podoviridae* (Supplementary Fig. S3). Moreover, the *S. aureus* PS66 *tarM* gene was sequenced and found to contain two non-synonymous point mutations (Q453K and A464E), which may compromise the TarM function and capacity to interfere with podovirus infection (Fig. 2a). Indeed, podovirus resistance of RN4220 ∆*tarM*, whose WTA contains only β-O-GlcNAc could be restored completely by complementation with a wild-type *tarM* but only partially by the mutated *tarM* (Fig. 2b). In addition, deletion of *tarS* in PS66 resulted in drastically reduced binding capacity of ΦP68 and rendered PS66 resistant to *Podoviridae* (Supplementary Fig. S4) suggesting that podovirus sensitivity of PS66 is linked to *tarS*-mediated β-O-GlcNAcylated WTA and to a strain-specific dysfunction of TarM.

**Figure 3:**
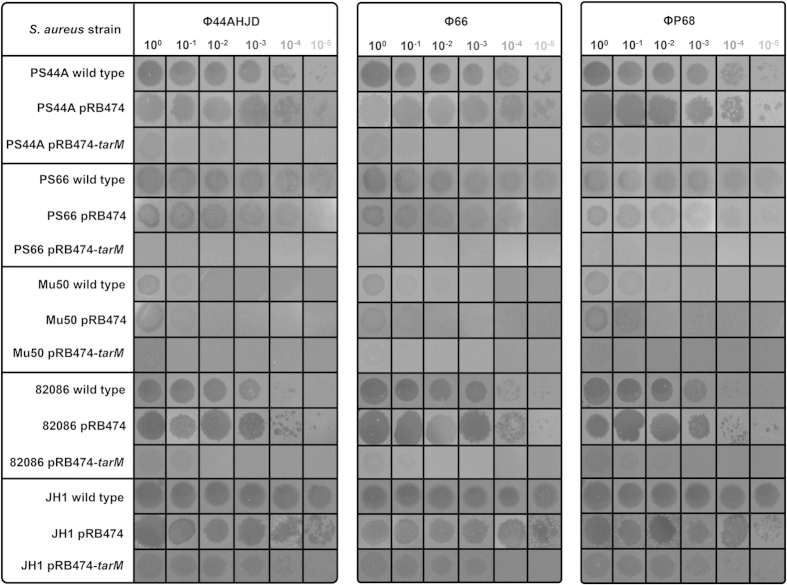
Ectopic expression of TarM protects podovirus-susceptible *S. aureus* against *Podoviridae*. The α-O-GlcNAc WTA glycosyltransferase TarM was ectopically expressed in various *tarM*-lacking and podovirus-susceptible *S. aureus* strains, and the phage susceptibility using a phage panel encompassing the lytic phages Φ44AHJD, Φ66 and ΦP68 (*Podoviridae*) was analyzed using a soft-agar overlay approach. Various dilutions of phage lysates, *S. aureus* wild type strains (*tarS* positive, but *tarM* negative (or encoding a mutated *tarM*, strain PS66)), and engineered strains expressing *tarM* (pRB474-*tarM*), or empty plasmid control (pRB474) are indicated. A representative experiment is shown.

Next, *tarM* was expressed in various podovirus*-*susceptible strains, including the Φ44AHJD and Φ66 propagation strains PS44A and PS66. Even at very high phage titers, expression of *tarM* rendered most susceptible strains completely resistant, confirming the importance of *tarM* in diminishing infection by staphylococcal *Podoviridae* (Fig. 3). In addition, the expression of a plasmid-born copy of *tarM* in strain PS66 also caused complete resistance to *Podoviridae*, further suggesting that the *tarM* gene of PS66 is most likely non-functional or less active (Fig. 3).

Thus, *Podoviridae* require β-O-GlcNAcylated WTA but cannot infect *S. aureus* with α-O-GlcNAcylated WTA.

### Tracking the evolution of TarM reveals an ancient origin in other staphylococcal species and vertical inheritance during *S. aureus* evolution

TarM is encoded outside of the *S. aureus* WTA gene clusters but does not appear to be encoded on a mobile genetic element^22^. Nevertheless, it is tempting to assume that it has been acquired by *S. aureus* at some point in evolution to interfere with podovirus infection.

**Figure 4:**
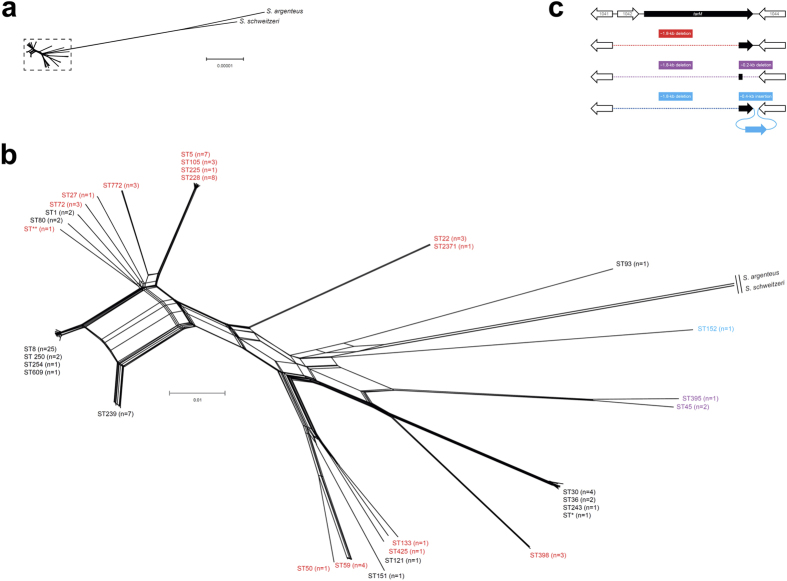
Phylogenetic distribution of *tarM* reveals an ancient origin in other staphylococci and vertical inheritance during *S. aureus* evolution. (**a,b**) Phylogenetic network representing the inferred relationship of 98 *S. aureus* strains and two closely related species, *S. argenteus* and *S. schweitzeri*. Strains are indicated by their multilocus sequence types (STs). ST* and ST** are single-locus variants of ST30 and ST1148, respectively. Strains encoding *tarM* are indicated in black, while strains lacking *tarM* are indicated in red, purple, and blue. (**c**) Genetic organization of the *tarM* region in *S. aureus*. The intact *tarM* region is shown in the upper cluster. Gene locus numbers refer to *S. aureus* strain COL (GenBank accession no. CP000046). Lower clusters indicate distinct deletion events involving *tarM*.

To track the emergence of TarM in *S. aureus*, the genome sequences of 98 *S. aureus* strains including those of most *S. aureus* laboratory test strains used in this study were obtained to infer their genetic relatedness (Fig. 4a,b). Of note, the presence of *tarM* in the most deeply branching *S. aureus* isolates MSHR1132 and FSA084, which were recently proposed as novel staphylococcal species *Staphylococcus argenteus* sp. nov. and *Staphylococcus schweitzeri* sp. nov.^23^, revealed that the presence of *tarM* is probably an ancient genetic trait of *S. aureus* (Fig. 4a). Still, homologues of *tarM* are also encoded by certain coagulase-negative staphylococci (e.g. specific *S. epidermidis* isolates) and even by non-staphylococcal species such as *Exiguobacterium oxidotolerans* and *Tetragenococcus halophilus*. Thus, the early evolution of *tarM* probably involved an ancient HGT event to the last common ancestor of contemporary *S. aureus* clones, further supported by the notion that *tarM* is flanked by a gene possibly related to conjugation (SACOL1042) (Fig. 4c). However, at a later stage of *S. aureus* evolution, different types of genetic rearrangements occurred in emerging phylogenetic branches such as CC5 or CC398, leading to a deletion of *tarM,* which rendered these podovirus-susceptible (Fig. 4c).

## Discussion

Staphylococcal *Podoviridae* infect an unusually wide panel of staphylococcal species but remain avirulent for certain *S. aureus* lineages probably as a result of the activity of the α-O-GlcNAc WTA glycosyltransferase TarM. In *tarM*-encoding strains, WTA polymers are probably glycosylated preferentially with α-O-GlcNAc, suggesting that TarM might be more active than TarS. Consequently, TarS-mediated β-O-GlcNAcylation is probably affected by the activity of TarM, thus preventing the adsorption and infection of *Podoviridae*. Even though it cannot be excluded that TarM potentially has additional and undiscovered functions, which may interfere with the adsorption or infection process, the drastically increased adsorption of ΦP68 in isogenic Δ*tarM* mutants suggests that α-O-GlcNAcylated WTA prevents the adsorption of *Podoviridae* to *S. aureus*. Nevertheless, one of the designated podovirus propagation strains (PS66) encoded both WTA glycosyltransferases suggesting that certain strains, despite encoding *tarM*, are potentially podovirus-susceptible. Here, TarM might be non-functional, dis-regulated, or mutated as observed in PS66, and cannot interfere with the activity of TarS. Nevertheless, this TarM-mediated phenomenon limits the host-range of *Podoviridae*, and thus, their therapeutic potential compared to other lytic staphylococcal phages such as *Myoviridae*.

Apart from this, it remains intriguing as to why certain strains and lineages have lost *tarM* during evolution to become podovirus-susceptible. Since both *S. aureus* and *S. aureus*-like species such as *S. schweitzeri* and *S. argenteus* encode *tarM* and *tarS*, and many human-associated *S. aureus* lineages have lost *tarM* during evolution, it can be assumed that *tarM* is probably not essential for continued adaptation to the human host. This is in agreement with the observation that both types of WTA O-GlcNAcylation, can mediate *S. aureus* binding to nasal epithelial cells and thus nasal colonization^24^. Also, human sera contain preferentially serum antibodies directed against TarS-dependent β-O-GlcNAcylated WTA, but not against TarM-mediated α-O-GlcNAcylated WTA^25^, suggesting that *tarM* may be down-regulated or less immunogenic than β-O-GlcNAcylated WTA during infections. It can be assumed that some *S. aureus* lineages did not eliminate *tarM* because WTA α-O-GlcNAcylation may provide *S. aureus* with a fitness benefit, whose basis remains to be identified in the future.

However, bearing *tarM* and TarM-mediated α-O-GlcNAcylated WTA protects *S. aureus* at least against the lytic activity of staphylococcal *Podoviridae* via a modification of the designated phage adsorption receptor. Such alterations of cell-surface structures serving as viral receptors are only one of many bacterial strategies to counteract phage infection and have also been described for other bacterial species^26^,^27^,^28^, but does not seem a general strategy of *S. aureus* to avoid phage adsorption and infection. Since other lytic staphylococcal phages such as *Myoviridae* are capable of infecting *tarM*-encoding *S. aureus* isolates, prevention of podovirus infection could be the result of a highly specific WTA-dependent mechanism in *S. aureus*, presumably as the result of adaptation to specific podovirus-rich environmental niches. In addition, altered phage-receptor binding proteins may easily change the host-range of *Podoviridae* to render *tarM*-bearing clones susceptible. Whereas bacterial phage resistance mechanisms such as CRISPR interference appear more efficient and widespread in prokaryotes these can also be bypassed, for example, by CRISPR-evading phages^29^ suggesting that host-virus interaction is a constantly evolving process.

## Methods

### Bacterial strains and growth conditions

All bacterial strains used in this study are listed in Supplementary Table S1. Unless otherwise noted, bacteria were grown in basic medium (BM) (1% tryptone, 0.5% yeast extract, 0.5% NaCl, 0.1% K_2_HPO_4_, 0.1% glucose) or lysogeny broth (Becton Dickinson) supplemented with appropriate antibiotics (Chlorampenicol 10 μg/ml, Ampicillin 100 μg/ml).

### Molecular genetic methods

*S. aureus* RN4220 and USA300 Δ*tarM*, Δ*tarS*, Δ*tarM* Δ*tarS*, and Δ*tagO* deletion mutants were described elsewhere^11^,^16^,^24^. For the construction of marker-less RN4220 Δ*srtA* mutant, or a PS66 Δ*tarS* mutant, the previously described *E. coli*/*S. aureus* shuttle vectors pIMAY or pKOR1 were used^30^,^31^. The corresponding primers are listed in Supplementary Table S2. Gene disruption by using pKOR1 or pIMAY was performed as described before^30^,^31^. Briefly, pKOR1-*tarS*, or pIMAY-*srtA* were isolated from an appropriate *E. coli* strain, and transformed into electrocompetent *S. aureus* RN4220 cells (and reisolated and transformed into PS66). Electroporation conditions were described before^11^. Knock-out plasmids were integrated onto the *S. aureus* genome at the permissive temperatures (37 °C, pIMAY; 43 °C, pKOR1) and in the presence of chloramphenicol (10 μg/ml). Counterselection was performed by using anhydrotetracycline (1 μg/ml). Resulting colonies were patched onto BM agar plates with and without chloramphenicol (10 μg/ml) and screened for plasmid loss. Gene deletion was confirmed via PCR in chloramphenicol-sensitive colonies.

For complementation studies (or *tarM* expression in *tarM*-lacking strain backgrounds), the previously described *E. coli*/*S. aureus* shuttle vector pRB474 was used^32^. pRB474-*tarM* (Q453K; A464E) has been described elsewhere (formerly pRB474-H-*tarM*)^15^.

### PCR-typing, sequencing, and multiple locus sequence typing (MLST)

For verification (and sequencing) of *tarM* and *tarS* in *S. aureus* genomes, PCR analysis using primers listed in Supplementary Table S2 was used. MLST typing of podovirus propagation strains PS44A, PS66 and P68 was performed as described previously using published primers^33^.

### Experiments with phages

All phages used in this study are listed in Supplementary Table S1. Phages were propagated on *S. aureus* strains P68 or RN4220 ∆*tarM* (Φ44AHJD, Φ66 and ΦP68), or RN4220 wild type (ΦK, Φ812) as described previously^34^. Briefly, the cognate *S. aureus* host strains were grown overnight at 37 °C in BM and diluted in phage-containing lysates (approximately 1 × 10^9^ plaque forming units (PfU) per milliliter; titrated on cognate host strains) to a final optical density OD 600 _nm_ of 0.4. Subsequently, CaCl_2_ was added to a final concentration of 4 mM. The bacteria/phage mixture was incubated for 30 min at 37 °C without agitation and afterwards for at least 3 h at 30 °C with mild agitation until complete lysis occurred. In order to remove cell debris, the lysate was centrifuged for 10 min (5,000 × g, 4 °C). Lysates were filter-sterilized (0.22 μm) and stored at 4 °C.

Phage susceptibility was analyzed as described elsewhere^17^. Briefly, a phage panel encompassing the broad host-range phages ΦK and Φ812 (*Myoviridae*), and three serogroup G phages Φ44AHJD, Φ66 and ΦP68 (*Podoviridae*) were used. 6 μl (approximately 1 × 10^9^ PfU/ml, or appropriate dilutions) of freshly propagated phage lysates were spotted onto bacterial lawns using the soft-agar overlay method described by Xia *et al.*^17^.

Phage adsorption to *S. aureus* strains was analyzed as described previously^17^. Briefly, the phage adsorption rate was analyzed using a multiplicity of infection (MOI) of 0.01 for phage ΦP68. Adsorption rate (%) was calculated by determining the number of unbound PfU in the supernatant and subtracting from the total number of input PfU as a ratio to the total number of input PfU.

### Phylogenetic analysis

The chromosomes of all *S. aureus* and *S. argenteus* and *S. schweitzeri* labelled as complete were obtained from GenBank (Supplementary Table S3) and aligned against the chromosome of *S. aureus* CC45 strain CA-347 (GenBank accession ID NC_021554) after identification and deletion of duplicated regions using MUMmer v 3.22^35^. The 98 publicly available genomes were aligned using MUMmer. Based on the identified core of ~1,9 Mb (67%) among all strains, a total of 312,427 SPNs was identified, from which the phylogenetic relationship was inferred using the NeighbourNets algorithm in SplitsTree v4.13.1^36^.

### RNA isolation and preparation

RNA was isolated as described previously^24^. Briefly, BM over-night cultures were diluted in BM. Bacteria were grown at 37 °C until lag, log, or stationary growth phases. Subsequently, bacteria were harvested and resolved in 1 ml TRIzol (Invitrogen/Life Technologies, Karlsruhe, Germany). Next, bacteria were mechanically disrupted by using a FastPrep24 homogenizer (MP Biomedicals) (2 cycles, 20 sec. at 6500 rpm each, with 0.5 ml Zirconia/Silica beads (0.1 mm in diameter; Carl-Roth, Karlsruhe, Germany)). Samples were either stored at −80 °C or subsequently used for further preparation. To each sample, 200 μl chloroform was added and samples were thoroughly mixed for 60 s, and incubated for 3 min at room temperature. Samples were centrifuged at 4 °C (12,000 × g, 15 min) and the supernatant was mixed with 500 μl isopropanol. Next, samples were incubated for 10 min at room temperature and centrifuged (12,000 × g, 30 min, 4 °C). Each pellet was washed with 500 μl ethanol (70%) and the sample was centrifuged (7,500 × g, 5 min, 4 °C). Finally, the pellet was air-dried and dissolved in 50 μl nuclease-free water. After incubation at 55 °C for 10 min, the sample was mixed well for 4 min. 5 μg RNA was digested with DNAse I (Roche) and stored at −80 °C.

### Quantitative real time PCR (qRT-PCR)

qRT-PCR was performed as described previously^24^. Briefly, RNA was transcribed into cDNA and qRT-PCR was performed according to the manufactures instructions using the Brilliant II SYBR© Green 1-Step Master Mix (Agilent). Relative quantifications were analyzed by using Roche’s LightCylcer480II. Transcription levels of target genes analyzed in this study were normalized against the expression of the housekeeping gene *gyrB*. All primers used for qRT-PCR are listed in Supplementary Table S2.

### Statistical analysis

Statistical analysis was performed using GraphPad Prism (GraphPad Software, Inc., La Jolla USA, Version 5.04). Statistically significant differences were calculated by using appropriate statistical methods as indicated. P values < 0.05 were considered significant.

## Additional Information

**How to cite this article**: Li, X. *et al.* An accessory wall teichoic acid glycosyltransferase protects *Staphylococcus aureus* from the lytic activity of *Podoviridae. Sci. Rep.* **5**, 17219; doi: 10.1038/srep17219 (2015).

## Supplementary Material

Supplementary Information

## Figures and Tables

**Table 1:** Lack of *tarM* in *S. aureus* correlates with susceptibility to *Podoviridae.*

*S. aureus* strain	Sequence type	*tarM*	*tarS*	Phage susceptibility^b^				
				*Myoviridae*		*Podoviridae*		
				ΦK	Φ812	Φ44AHJD	Φ66	ΦP68
MW2	1	+	+	+	+	—	—	—
Mu50	5	—	+	(+)	+	+	+	+
USA300	8	+	+	+	+	—	—	—
NRS184	22	—	+	(+)	+	+	+	+
P68	25	—	+	(+)	(+)	+	+	+
UAMS-1	30	+	+	+	+	—	—	—
PS66	39	+	+	+	+	+	+	+
USA600	45	—	+	(+)	—	—	—	—
JH1	105	—	+	+	+	+	+	+
ED133	133	—	—	+	(+)	—	—	—
RF122	151	+	+	+	+	+	+	+
605	239	+	+	(+)	(+)	—	—	—
Col	250	+	+	+	+	—	—	—
PS187^a^	395	—	—	+	+	—	—	—
82086	398	—	+	+	+	+	+	+
PS44A	707	—	+	+	+	+	+	+

^a^PS187 synthesizes a poly-glycerol phosphate WTA type modified with α-O-N-Acetylgalactosamine (mediated by the ST395-specific WTA glycosyltransferase TagN^12^).

^b^Phage susceptibility was analyzed via soft agar overlay method. Phage susceptibility (+) or resistance is indicated (—). Diminished plaque formation (ΦK, Φ812) observed for strains Mu50, NRS184, P68, USA600, ED133, and 605 is indicated with a bracketed plus symbol ((+)).
