# Supplementary material for: An accessory wall teichoic acid glycosyltransferase protects Staphylococcus aureus from the lytic activity of Podoviridae
Source: Sci Rep. 2015 Nov 24;5:17219. doi: 10.1038/srep17219 (PMC4667565; doi:10.1038/srep17219)
Supplement: Supplementary Information [file srep17219-s1.pdf]

Supplementary Material

An accessory wall teichoic acid glycosyltransferase protects *Staphylococcus aureus* from the lytic activity of *Podoviridae*

Xuehua Li[1,2], David Gerlach[1,2], Xin Du[1,2], Jesper Larsen[3], Marc Stegger[3,4], Petra Kühner[1,2], Andreas Peschel[1,2], Guoqing Xia[1,2,5],Volker Winstel[1,2,6*]

[1] Infection Biology, Interfaculty Institute of Microbiology and Infection Medicine, University of Tübingen, Auf der Morgenstelle 28, 72076 Tübingen, Germany

[2] German Center for Infection Research (DZIF), partner site Tübingen, 72076 Tübingen, Germany

[3] Microbiology and Infection Control, Statens Serum Institute, Artillerivej 5, 2300 Copenhagen, Denmark

[4] Pathogen Genomics Division, Translational Genomics Research Institute, 3051 W Shamrell Blvd, Flagstaff, 86001 Arizona, USA

[5] Institute of Inflammation & Repair, The University of Manchester, Manchester, United Kingdom

[6] Current address: Department of Microbiology, University of Chicago, 920 East 58th Street, Chicago, Illinois 60637, USA

* Correspondence to: e-mail: vwinstel@bsd.uchicago.edu

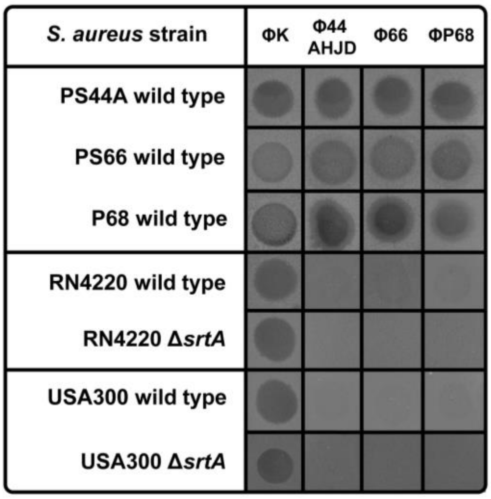

Supplementary Figure S1 – Impact of peptidoglycan-anchored surface proteins on host-specificity of *Podoviridae*. *S. aureus* RN4220 and USA300 susceptibility to the broad host- range lytic phage ΦK (*Myoviridae*), and to the lytic phages Φ44AHJD, Φ66 and ΦP68 (*Podoviridae*) was analyzed using a soft-agar overlay approach. *S. aureus* podovirus propagation strains (PS44A, PS66, and P68), *S. aureus* wild type, and mutants lacking peptidoglycan-anchored surface proteins (Δ*srtA*) are indicated. A representative experiment is shown.

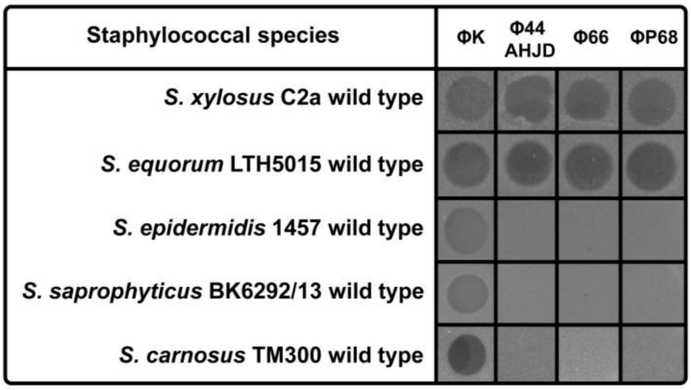

Supplementary Figure S2 – Susceptibility of selected staphylococcal species to *Podoviridae*. Susceptibility of selected staphylococcal species to the broad host-range lytic phage ΦK (*Myoviridae*), and to the lytic phages Φ44AHJD, Φ66 and ΦP68 (*Podoviridae*) was analyzed using a soft-agar overlay approach. A representative experiment is shown.

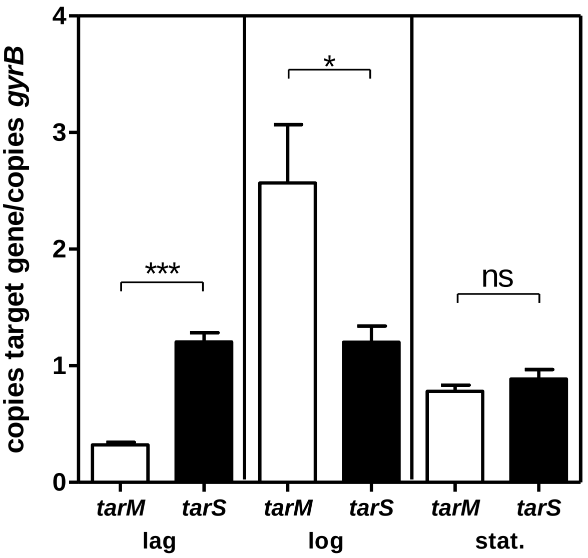

Supplementary Figure S3 – qRT-PCR analysis of WTA glycosyltransferases in Φ66 propagation strain PS66. mRNA was isolated from lag-, log-, or stationary (stat.)-phase-grown bacteria. Values are given as means and standard deviations (SD; n = 3). Statistically significant differences calculated using an unpaired two-tailed Student's t test are indicated as follows: ns (not significant), P > 0.05; *, P < 0.05; ***, P < 0.001.

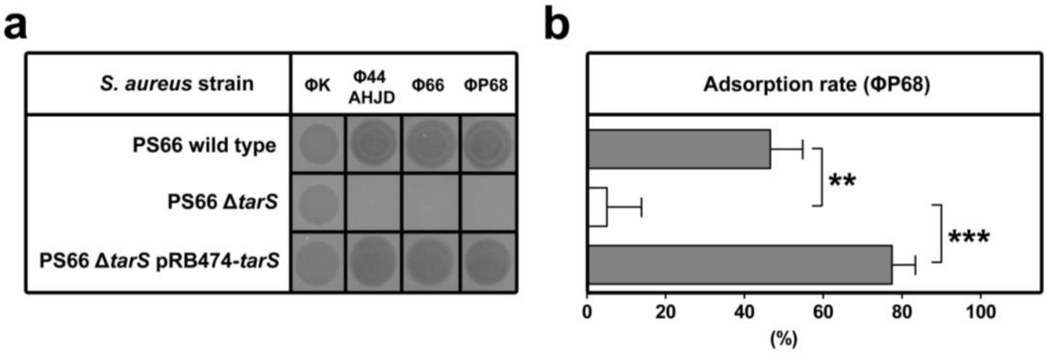

Supplementary Figure S4 – Lack of the β-O-GlcNAc WTA glycosyltransferase TarS renders Φ66 propagation strain PS66 resistant to *Podoviridae*. (a) *S. aureus* PS66 susceptibility to the broad host-range lytic phage ΦK (*Myoviridae*), and to the lytic phages Φ44AHJD, Φ66 and ΦP68 (*Podoviridae*) was analyzed using a soft-agar overlay approach. A representative experiment is shown. **(b)** Podovirus ΦP68 adsorption rate (%) to *S. aureus* PS66 variants. *S. aureus* wild type, the strain lacking WTA β-O-GlcNAcylation (Δ*tarS*), and the complemented Δ*tarS* mutant (Δ*tarS* pRB474-*tarS*) are indicated. Values are given as means and standard deviations (SD, n = 3). Statistical significant differences calculated by one-way ANOVA with Bonferroni's multiple comparison test are indicated: not significant (ns), P > 0.05; *, P < 0.05, **, P < 0.01; ***, P < 0.001; ****, P < 0.0001.

Supplementary Table S1 - Bacterial strains and phages used in this study

Bacterial strain or phage	Description	Source
E. coli TOP10	One Shot® TOP10 chemically competent *E. coli*	Invitrogen
E. coli DB 3.1 pKOR1	DB3.1 strain, bears pKOR1 plasmid	[30]
E. coli DC10B pIMAY	DH10B Δ*dcm*; Dam methylation only, bears pIMAY plasmid	[31]
S. aureus RN4220	Wild type, deficient in restriction, capsule, and prophage	[37]
S. aureus RN4220 Δ*tagO*	RN4220 Δ*tagO*	[17]
S. aureus RN4220 Δ*tarM*	RN4220 Δ*tarM*	[11]
S. aureus RN4220 Δ*tarS*	RN4220 Δ*tarS*	[11]
S. aureus RN4220 Δ*tarM* Δ*tarS*	RN4220 Δ*tarM* Δ*tarS*	[11]
S. aureus RN4220 Δ*tarM* Δ*tarS* pRB474-*tarM*	RN4220 Δ*tarM* Δ*tarS* complemented with *tarM*	[11]
S. aureus RN4220 Δ*tarM* Δ*tarS* pRB474-*tarS*	RN4220 Δ*tarM* Δ*tarS* complemented with *tarS*	[11]
S. aureus RN4220 Δ*srtA*	RN4220 Δ*srtA*	This study
S. aureus USA300	Wild type, NRS384, ST8, CA-MRSA	NARSA strain collection
S. aureus USA300 Δ*tagO*	USA300 Δ*tagO*	[24]
S. aureus USA300 Δ*tarM*	USA300 Δ*tarM*	[24]
S. aureus USA300 Δ*tarS*	USA300 Δ*tarS*	[16]
S. aureus USA300 Δ*tarM* Δ*tarS*	USA300 Δ*tarM* Δ*tarS*	[24]
S. aureus USA300 Δ*tarM* Δ*tarS* pRB474-*tarM*	USA300 Δ*tarM* Δ*tarS* complemented with *tarM*	[24]
S. aureus USA300 Δ*tarM* Δ*tarS* pRB474-*tarS*	USA300 Δ*tarM* Δ*tarS* complemented with *tarS*	[24]
S. aureus USA300 Δ*srtA*	USA300 Δ*srtA*	[24]
S. aureus PS44A	Wild type, PS44A, NCTC 8369, ST707, designated propagation strain for Φ44AHJD	NCTC collection
S. aureus PS66	Wild type, PS66, NCTC 8288, ST39, designated propagation strain for Φ66	Obtained from Udo Bläsi, Vienna
S. aureus PS66 Δ*tarS*	PS66 Δ*tarS*	This study
S. aureus PS66 Δ*tarS* pRB474-*tarS*	PS66 Δ*tarS* complemented with *tarS*	This study
S. aureus P68	Wild type, P68, ST25, designated propagation strain for ΦP68	Obtained from Udo Bläsi, Vienna
S. aureus RF122	Wild type, bovine isolate, ST151	[38]
S. aureus USA600	Wild type, NRS22, ST45	NARSA strain collection
S. aureus ED133	Wild type, ovine isolate, ST133	[39]
S. aureus Col	Wild type, clinical isolate, ST250	[40]
S. aureus PS187	Wild type, PS187, ST395	[41]
S. aureus MW2	Wild type, MW2, ST1	[42]
S. aureus Mu50	Wild type, Mu50, ST5	[43]
S. aureus NRS184	Wild type, NRS184, ST22	NARSA strain collection
S. aureus JH1	Wild type, JH1, ST105	[44]
S. aureus 605	Wild type, 605, ST239	[45]
S. aureus 82086	Wild type, 82086, ST398	[24]
S. aureus 82086 pRB474-*tarM*	Wild type, 82086, bears pRB474-*tarM*	This study
S. aureus 82086 pRB474	Wild type, 82086, bears pRB474	This study
S. aureus Mu50 pRB474-*tarM*	Wild type, Mu50, bears pRB474-*tarM*	This study
S. aureus Mu50 pRB474	Wild type, Mu50, bears pRB474	This study
S. aureus JH1 pRB474-*tarM*	Wild type, JH1, bears pRB474-*tarM*	This study
S. aureus JH1 pRB474	Wild type, JH1, bears pRB474	This study

S. aureus PS44A pRB474-*tarM*	Wild type, PS44A, bears pRB474-*tarM*	This study
S. aureus PS44A pRB474	Wild type, PS44A, bears pRB474	This study
S. aureus PS66 pRB474-*tarM*	Wild type, PS66, bears pRB474-*tarM*	This study
S. aureus PS66 pRB474	Wild type, PS66, bears pRB474	This study
S. xylosus C2a	Wild type, human skin isolate, DSM20267	[46]
S. equorum LTH5015	Wild type	Obtained from Friedrich Götz, Tuebingen
S. epidermidis 1457	Wild type, clinical isolate	[47]
S. saprophyticus BK6292/13	Wild type, clinical isolate	Obtained from Holger Rohde, Hamburg
Phage ΦK	*Myoviridae*, Serogroup D	[48]
Phage Φ812	*Myoviridae*, Serogroup D	[19]
Phage Φ44AHJD	*Podoviridae*, Serogroup G	Obtained from Udo Bläsi, Vienna
Phage Φ66	*Podoviridae*, Serogroup G	Obtained from Udo Bläsi, Vienna
Phage Φ68	*Podoviridae*, Serogroup G	Obtained from Udo Bläsi, Vienna

Supplementary Table S2 – Oligonucleotides used in this study

Primer	Sequence	Application	Reference
tarM-up	ATGAAAAAAATATTTATGATGGTACATGAGTTAGA	PCR-typing *tarM*	[24]
tarM-dn	TTAGCTATTGAAAAGATTTAACCATTTTTCTAATA	PCR-typing *tarM*	[24]
tarS-up	ATGATGAAATTTTCAGTAATAGTTCCAACATACAA	PCR-typing *tarS*	[24]
tarS-dn	TTATTTTAGCGAGTAAGTCATATGTGCAGT	PCR-typing *tarS*	[24]
tarM-for2	TAATGCTAATAATGGTGCTG	qRT-PCR	[16]
tarM-rev2	GGTCCATCACAAATCATAAT	qRT-PCR	[16]
tarS-for2	CACGAAACAAGAAGCACA	qRT-PCR	[16]
tarS-rev2	TGATTACCAACACGCACT	qRT-PCR	[16]
gyrBF	GGTGGCGACTTTGATCTAGC	qRT-PCR	[49]
gyrBRv	TTATACAACGGTGGCTGTGC	qRT-PCR	[49]

Supplementary Table S3 – Genome sequences used for phylogenetic analysis

Species	Strain	Accession	Total length	ST	*tarM*	*tarS*
S. aureus	MW2	BA000033	2820462 bp	1	+	+
S. aureus	MSSA476	BX571857	2799802 bp	1	+	+
S. aureus	Mu3	AP009324	2880168 bp	5	-	+
S. aureus	Mu50	BA000017	2878529 bp	5	-	+
S. aureus	N315	BA000018	2814816 bp	5	-	+
S. aureus	ED98	CP001781	2824404 bp	5	-	+
S. aureus	502A	CP007454	2764699 bp	5	-	+
S. aureus	ECT-R 2	FR714927	2729540 bp	5	-	+
S. aureus	M0628	KB821506	2828436 bp	5	-	+
S. aureus	NCTC 8325	CP000253	2821361 bp	8	+	+
S. aureus	USA300_FPR3757	CP000255	2872769 bp	8	+	+
S. aureus	USA300_TCH1516	CP000730	2872915 bp	8	+	+
S. aureus	VC40	CP003033	2692570 bp	8	+	+
S. aureus	USA300-ISMMS1	CP007176	2921008 bp	8	+	+
S. aureus	2395 USA500	CP007499	2955646 bp	8	+	+
S. aureus	M1216	CP007670	2896143 bp	8	+	+
S. aureus	CA15	CP007674	2839253 bp	8	+	+
S. aureus	UA-S391_USA300	CP007690	2872916 bp	8	+	+
S. aureus	29b_MRSA (ATCC BAA-1680)	CP010295	2872768 bp	8	+	+
S. aureus	31b_MRSA (ATCC BAA-1680)	CP010296	2872779 bp	8	+	+
S. aureus	33b (ATCC BAA-1680)	CP010297	2872764 bp	8	+	+
S. aureus	26b_MRSA (ATCC BAA-1680)	CP010298	2872779 bp	8	+	+
S. aureus	25b_MRSA (ATCC BAA-1680)	CP010299	2872781 bp	8	+	+
S. aureus	27b_MRSA (ATCC BAA-1680)	CP010300	2872771 bp	8	+	+
S. aureus	DSM 20231	CP011526	2755072 bp	8	+	+
S. aureus	M1	HF937103	2864125 bp	8	+	+
S. aureus	W48872	KK022849	2859671 bp	8	+	+
S. aureus	T87526	KK027252	2867276 bp	8	+	+
S. aureus	F26088	KK029104	2859631 bp	8	+	+
S. aureus	W15997	KK032093	2860093 bp	8	+	+
S. aureus	M49474	KK072349	2859938 bp	8	+	+
S. aureus	T36111	KK073475	2859914 bp	8	+	+
S. aureus	T83543	KK095312	2860767 bp	8	+	+
S. aureus	NCTC8532	LN831049	2709282 bp	8	+	+
S. aureus	H-EMRSA-15	CP007659	2846320 bp	22	-	+
S. aureus	71A_S11	CP010940	2756431 bp	22	-	+
S. aureus	HO 5096 0412	HE681097	2832299 bp	22	-	+

S. aureus	RKI4	CP011528	2725654 bp	27	-	+
S. aureus	55/2053	CP002388	2756919 bp	30	+	+
S. aureus	FORC_001	CP009554	2886017 bp	30	+	+
S. aureus	UAMS-1	JTJK00000000	2763963 bp	30	+	+
S. aureus	ILRI_Eymole1/1	LN626917	2874302 bp	30	+	+
S. aureus	MRSA252	BX571856	2902619 bp	36	+	+
S. aureus	M0513	KB821413	2932850 bp	36	+	+
S. aureus	CA-347	CP006044	2850503 bp	45	-	+
S. aureus	NRS22	JYAG00000000	2922078 bp	45	-	+
S. aureus	6850	CP006706	2736560 bp	50	-	+
S. aureus	M013	CP003166	2788636 bp	59	-	+
S. aureus	SA957	CP003603	2789538 bp	59	-	+
S. aureus	SA40	CP003604	2728308 bp	59	-	+
S. aureus	SA268	CP006630	2833899 bp	59	-	+
S. aureus	TMUS2126	AP014652	2770164 bp	72	-	+
S. aureus	TMUS2134	AP014653	2770164 bp	72	-	+
S. aureus	CN1	CP003979	2751266 bp	72	-	+
S. aureus	11819-97	CP003194	2846546 bp	80	+	+
S. aureus	NCTC13435	LN831036	2797452 bp	80	+	+
S. aureus	JKD6159	CP002114	2811435 bp	93	+	+
S. aureus	JH9	CP000703	2906700 bp	105	-	+
S. aureus	JH1	CP000736	2906507 bp	105	-	+
S. aureus	FCFHV36	CP011147	2849811 bp	105	-	+
S. aureus	93b_S9	CP010952	2788353 bp	121	+	+
S. aureus	ED133	CP001996	2832478 bp	133	-	-
S. aureus	RF122	AJ938182	2742531 bp	151	+	+
S. aureus	SA17_S6	CP010941	2672185 bp	152	-	+
S. aureus	04-02981	CP001844	2821452 bp	225	-	+
S. aureus	10388	HE579059	2759510 bp	228	-	+
S. aureus	10497	HE579061	2759512 bp	228	-	+
S. aureus	15532	HE579063	2759883 bp	228	-	+
S. aureus	16035	HE579065	2759835 bp	228	-	+
S. aureus	16125	HE579067	2759457 bp	228	-	+
S. aureus	18341	HE579069	2759473 bp	228	-	+
S. aureus	18412	HE579071	2759263 bp	228	-	+
S. aureus	18583	HE579073	2759328 bp	228	-	+
S. aureus	JKD6008	CP002120	2924344 bp	239	+	+
S. aureus	T0131	CP002643	2913900 bp	239	+	+
S. aureus	Bmb9393	CP005288	2980548 bp	239	+	+
S. aureus	Z172	CP006838	2987966 bp	239	+	+
S. aureus	XN108	CP007447	3052055 bp	239	+	+
S. aureus	Gv69	CP009681	3046210 bp	239	+	+
S. aureus	TW20	FN433596	3043210 bp	239	+	+

S. aureus	ATCC 25923	CP009361	2778854 bp	243	+	+
S. aureus	Col	CP000046	2809422 bp	250	+	+
S. aureus	NRS 100	CP007539	2823087 bp	250	+	+
S. aureus	Newman	AP009351	2878897 bp	254	+	+
S. aureus	PS187	ARPA00000000	2781079 bp	395	-	-
S. aureus	SO385	AM990992	2872582 bp	398	-	+
S. aureus	71193	CP003045	2715000 bp	398	-	+
S. aureus	08BA02176	CP003808	2782313 bp	398	-	+
S. aureus	LGA251	FR821779	2750834 bp	425	-	+
S. aureus	M0831	KB821688	2957781 bp	609	+	+
S. aureus	DAR4145	CP010526	2860508 bp	772	-	+
S. aureus	144_S7	CP010943	2730860 bp	772	-	+
S. aureus	79_S10	CP010944	2726524 bp	772	-	+
S. aureus	SASCBU26	CDLR00000000	2862578 bp	2371	-	+
S. aureus	TCH60	CP002110	2802675 bp	*	+	+
S. aureus	M1216	KB822075	2793375 bp	**	-	+
S. argenteus	MSHR1132	FR821777	2762785 bp	1850	+	+
S. schweitzeri	FSA084	CCEL00000000	2748405 bp	2022	+	+

Supplementary References

37 Kreiswirth, B. N. *et al.* The toxic shock syndrome exotoxin structural gene is not detectably transmitted by a prophage. *Nature* **305**, 709-712 (1983).

38 Fitzgerald, J. R., Hartigan, P. J., Meaney, W. J. & Smyth, C. J. Molecular population and virulence factor analysis of Staphylococcus aureus from bovine intramammary infection. *Journal of applied microbiology* **88**, 1028-1037 (2000).

39 Ben Zakour, N. L. *et al.* Genome-wide analysis of ruminant Staphylococcus aureus reveals diversification of the core genome. *Journal of bacteriology* **190**, 6302-6317, doi:10.1128/JB.01984-07 (2008).

40 Dyke, K. G., Jevons, M. P. & Parker, M. T. Penicillinase production and intrinsic resistance to penicillins in Staphylococcus aures. *Lancet* **1**, 835-838 (1966).

41 Asheshov, E. A. & Jevons, M. P. The effect of heat on the ability of a host strain to support the growth of a Staphylococcus phage. *Journal of general microbiology* **31**, 97-107 (1963).

42 Centers for Disease, C. & Prevention. Four pediatric deaths from community-acquired methicillin-resistant Staphylococcus aureus - Minnesota and North Dakota, 1997-1999. *MMWR. Morbidity and mortality weekly report* **48**, 707-710 (1999).

43 Kuroda, M. *et al.* Whole genome sequencing of meticillin-resistant Staphylococcus aureus. *Lancet* **357**, 1225-1240 (2001).

44 Mwangi, M. M. *et al.* Tracking the in vivo evolution of multidrug resistance in Staphylococcus aureus by whole-genome sequencing. *Proceedings of the National Academy of Sciences of the United States of America* **104**, 9451-9456, doi:10.1073/pnas.0609839104 (2007).

45 Li, M. *et al.* MRSA epidemic linked to a quickly spreading colonization and virulence determinant. *Nature medicine* **18**, 816-819, doi:10.1038/nm.2692 (2012).

46 Schleifer, K. H. & Kloos, W. E. Isolation and Characterization of Staphylococci from Human Skin .1. Amended Descriptions of Staphylococcus-Epidermidis and Staphylococcus-Saprophyticus and Descriptions of 3 New Species - Staphylococcus-Cohnii, Staphylococcus-Haemolyticus, and Staphylococcus-Xylosus. *International Journal of Systematic Bacteriology* **25**, 50-61 (1975).

47 Mack, D., Siemssen, N. & Laufs, R. Parallel induction by glucose of adherence and a polysaccharide antigen specific for plastic-adherent Staphylococcus epidermidis: evidence for functional relation to intercellular adhesion. *Infection and immunity* **60**, 2048-2057 (1992).

48 O'Flaherty, S. *et al.* Potential of the polyvalent anti-Staphylococcus bacteriophage K for control of antibiotic-resistant staphylococci from hospitals. *Applied and environmental microbiology* **71**, 1836-1842, doi:10.1128/AEM.71.4.1836-1842.2005 (2005).

49 Goerke, C. *et al.* Role of Staphylococcus aureus global regulators sae and sigmaB in virulence gene expression during device-related infection. *Infection and immunity* **73**, 3415-3421, doi:10.1128/IAI.73.6.3415-3421.2005 (2005).
